# The right to mental health protection and its status in European Union primary law

**DOI:** 10.1192/j.eurpsy.2025.588

**Published:** 2025-08-26

**Authors:** M. Burdzik

**Affiliations:** 1 Dr. Krzysztof Czuma’s Psychiatric Center; 2 Institute of Law, University of Silesia, Katowice, Poland

## Abstract

**Introduction:**

Mental health is one of the fundamental personal values of every human being. Thus its protection should be one of the primary obligations of every state. To ensure common standards in aforementioned matter, it is necessary to exist an universal (supranational) grounds and rules for the protection of mental health, as well as the protection of people with mental disorders rights. Especially that, mental disorders are found all over the world, and in an age of globalisation and migration, borders are only a legal construct. In the Western European axionormative order, one of the factors shaping common normative standards is European Union law.

**Objectives:**

The aim of this study is to identify the norms of European Union primary law from which the right to mental health protection can be derived (directly or indirectly), with a subsequent assessment of their adequacy.

**Methods:**

The study is based on a dogmatic analysis of the primary legislation of the European Union (i.a. the founding Treaties or the Charter of Fundamental Rights of the EU - CFR) with analysis of the relevant literature of the subject and jurisprudence of the Court of Justice of the European Union.

**Results:**

No provision of EU primary law explicitly statutes a ‘right to mental health protection’. Currently, this right can be derived indirectly from norms relating to its particular aspects - e.g. the protection of mental integrity (Article 3 of CFR or Article 165(2) of the Treaty on the Functioning of the European Union - TFEU), the removal of threats to mental health (Article 168(1) TFEU) or the prohibition of discrimination on grounds of disability (Article 10 TFEU).

**Conclusions:**

The current regulations are incomplete and therefore inadequate. The protection of mental integrity or the fight against discrimination are necessary for mental health (well-being) of entities, but do not fully cover the latter concept. The strengthening of the legal position of people with mental disorders requires the introduction into EU legislation of a comprehensive regulation that statutes the right to mental health protection, together with mechanisms for its enforcement against state authorities.

**Disclosure of Interest:**

None Declared

